# Sex-Related Differences in Dilated Cardiomyopathy with a Focus on Cardiac Dysfunction in Oncology

**DOI:** 10.1007/s11886-020-01377-z

**Published:** 2020-08-08

**Authors:** Domenico D’Amario, Massimiliano Camilli, Stefano Migliaro, Francesco Canonico, Mattia Galli, Alessandra Arcudi, Rocco Antonio Montone, Josip Andjelo Borovac, Filippo Crea, Gianluigi Savarese

**Affiliations:** 1 Dipartimento di Scienze Cardiovascolari, Fondazione Policlinico Universitario A. Gemelli IRCCS, Rome, Italy; 2 Dipartimento di Scienze Cardiovascolari, Università Cattolica del Sacro Cuore, Rome, Italy; 3 Department of Pathophysiology, University of Split, Split, Croatia; 4 Cardiology Division, Department of Medicine, Karolinska Institutet, Stockholm, Sweden

**Keywords:** Dilated cardiomyopathy, Cardio-oncology, Sex difference, Heart failure

## Abstract

**Purpose of Review:**

The aim of this report is to describe the main aspects of sex-related differences in non-ischemic dilated cardiomyopathies (DCM), focusing on chemotherapy-induced heart failure (HF) and investigating the possible therapeutic implications and clinical management applications in the era of personalized medicine.

**Recent Findings:**

In cardio-oncology, molecular and multimodality imaging studies confirm that sex differences do exist, affecting the therapeutic cardioprotective strategies and, therefore, the long-term outcomes. Interestingly, compelling evidences suggest that sex-specific characteristics in drug toxicity might predict differences in the therapeutic response, most likely due to the tangled interplay between cancer and HF, which probably share common underlying mechanisms.

**Summary:**

Cardiovascular diseases show many sex-related differences in prevalence, etiology, phenotype expression, and outcomes. Complex molecular mechanisms underlie this diverse pathological manifestations, from sex-determined differential gene expression to sex hormone interaction with their receptors in the heart. Non-ischemic DCM is an umbrella definition that incorporates several etiologies, including chemotherapy-induced cardiomyopathies. The role of sex as a risk factor for cardiotoxicity is poorly explored. However, understanding the various features of disease manifestation and outcomes is of paramount importance for a prompt and tailored evaluation.

## Introduction: Dilated Cardiomyopathy with a Focus on Cardio-Oncology

Dilated cardiomyopathy (DCM) is a general definition used to describe the cardiac effects of a broad spectrum of diseases, characterized by the presence of left- or bi-ventricular dilatation and systolic dysfunction in the absence of coronary artery disease, hypertension, valvular or congenital disease [1]*.* However, international societies adopt heterogeneous classification of this entity: the American Heart Association distinguishes among genetic, mixed, or acquired causes while the European Society of Cardiology categorize into genetic or non-genetic variants [2, 3].

Recognized etiologies include idiopathic forms, which are still the most common [4], and familial forms, with extreme heterogeneity in the number and type of both structural and functional genes involved, which up to 35% of cases [5]. Infective forms are instead mainly sustained by viruses (e.g., Adenovirus spp., Coronavirus spp., Coxsackievirus spp., influenza virus, Herpesviridae, Hepatoviridae, Parvovirus), followed by bacteria and fungi, protozoa, or helminths [6]*.* Human immunodeficiency virus (HIV)–associated cardiomyopathy was firstly described in the mid-1980s and characterized by LV enlargements and systolic dysfunction, due to autoimmune reactions, myocarditis, nutritional deficiencies, or severe immunosuppression. In this subgroup of patients, the occurrence of DCM had a detrimental impact on median survival, until the introduction of antiretroviral therapy (ART) that on the one hand improved prognosis, but on the other one may contribute to myocardial dysfunction [7]*.* Among infectious disorders bringing to DCM, Chagas disease is worth noting, determined by the protozoan *Trypanosoma cruzi* transmission. It is responsible for a large cohort of cardiological manifestations, among which symptomatic dilated Chagas cardiomyopathy represents the most advanced stage of disease [8]*.* Traditionally considered as a tropical disease (Brazil, Argentina, and Bolivia have the largest number of individuals affected), Chagas disease now interests thousands of residents of the USA and other traditionally nonendemic areas [8]*.* Autoimmune causes [9], metabolic or endocrine dysfunction (e.g., Cushing disease, hypo/hyperthyroidism, mitochondrial diseases), and neuromuscular diseases (e.g., various forms of muscular dystrophy, Friedreich ataxia) [10] are rarer yet not negligible causes. Peripartum cardiomyopathy is a potentially life-threatening pregnancy-associated disease that occurs infrequently and is marked by transient left ventricular dysfunction and heart failure (HF) [11]. Lastly, toxic damage secondary to abuse substances (mainly alcohol, cannabis, cocaine), or exposure to some pharmacological agents, as many anti-blastic drugs, is a major cause of DCM [12]*.*

Incidence of DCM in post-mortem studies was reviewed over time with recent data estimating a number of more than 1 on 250 individuals affected [4, 13], with a global prevalence of 2.5 million people up to 2015 [14], while the latest data on mortality show an incidence of 5.9 deaths per 100,000 people [15]. Looking at the recent analyses from the landmark PARADIGM-HF trial, DCM accounts for 19% of all HF with reduced ejection fraction (HFrEF) cases [16]*.* Deaths arise mainly from pump failure, accounting for 70% of cases, while arrhythmias accounted for the remaining 30% [17]. Significant epidemiological differences have been described in relation to geographical location and ethnicity. DCM is the leading cause of HFrEF in Asia Pacific region (28%), followed by Latin America (21%) and Central/Eastern Europe and North America (14%) [16]. Within DCM patients, the black ethnicity seems to be associated with a higher risk of mortality. Compared with other forms of HF, DCM patients tend to be 5–10 years younger and up to three times more often male, with less comorbidities [18–20]*.*

In recent years, the number of patients diagnosed with DCM secondary to anti-blastic drugs is constantly increasing given both the aging of the general population and the increase in cancer incidence and survival: it is estimated that by 2040 the number of Americans with a history of cancer will increase to more than 26 millions [21]*.* In this setting, DCM can be the ending phase of a pathological process that is usually defined more broadly as chemotherapy-related cardiac dysfunction (CRCD). It is reported that CRCD can affect up to 10% of cancer survivors [22], even though incidence is variable according to the definition used. The heterogeneity of definitions adopted by different societies is summarized in Table 1 [23, 24]. CRCD progresses to end-stage HF in 2–3% of cases according to retrospective studies [22, 25–28]; up to 2.5% of patients requiring LVAD or transplantation have CRCD in UNOS or INTERMACS registries [22, 25–28]. Both DCM in general and CRCD in particular are influenced by sex in terms of clinical characteristics and outcomes.

**Table 1:** Synoptic overview of different definitions adopted for cardio-oncology

Society	Definition
The America Society of Echocardiography (ASE) and European Association of Cardiovascular Imaging (EACVI)	LVEF fall by > 10% to absolute EF < 53% confirmed on subsequent imaging performed 2 to 3 weeks after initial measurements
US Food and Drug Administration (FDA)	Doxorubicin-mediated cardiotoxicity was defined as either 1. > 20% absolute decline in LVEF 2. > 10 decrease in LVEF to less than the lower limit of normal or absolute value less than 45%
Cardiac Review and Evaluation Committee in Trastuzumab trials	Trastuzumab cardiomyopathy was defined as: > 10% decline in LVEF without symptoms > 5% decrease in symptomatic patients to a final LVEF below 55%
Herceptin Adjuvant trial (HERA)	LVEF decline by at least 10% from baseline to less than 50%
The Breast Cancer International Research Group (BCIRG)	> 10% reduction in LVEF from baseline assessment
The National Cancer Institute (NCI)	Introduces the Common Terminology Criteria for Adverse Events (CTCAE) that defines left ventricular dysfunction and HF based on severity into grades 1–5 -Grade 1: asymptomatic elevation in biomarker or imaging abnormality -Grades 2 and 3: symptoms with mild or moderate exertion -Grade 4: severe, life-threatening symptoms requiring hemodynamic support -Grade 5: death
European Society of Cardiology (ESC)	> 10% reduction in LVEF from baseline to < 50%
European Society of Medical Oncology (ESMO)	Symptomatic decline in LVEF of at least 5 to < 55% or asymptomatic decline in LVEF of at least 10 to < 55%

*HF* heart failure, *LVEF* left ventricle ejection fraction

Sex and gender are two separate but intertwined terms for evaluation and analyses of men and women. Sex is associated with biologic functions and gender goes beyond biology and is associated with culture and the environment. In medicine, it is difficult to isolate the two concepts because they interact and become tangled together.

The purpose of this review is to outline the main aspects of sex-related differences in non-ischemic dilated cardiomyopathies (DCM), focusing on chemotherapy-induced heart failure and investigating possible management applications in the era of personalized medicine.

## Mechanisms of DCM

DCM is a wide, unspecific definition which encompasses multiple diseases and pathophysiological processes, leading to the same clinical phenotype, as a result of complex interaction between environment and genetic predisposition.

The rising number of cancer survivors uncovered the need that all healthcare providers involved in the care of these patients should be fully aware of the impact of adverse cardiovascular effects on the survival when new or pre-existing cardiovascular diseases are present. Until now, the main focus of cardio-oncology has been the prevention and treatment of cardiotoxic effects of chemotherapeutic agents. Due to the development of novel anticancer targeted lines of therapy, this approach is gaining again the central stage in the management of these patients. However, a new captivating area of cardio-oncology is highlighting the relevance of pre-existing, or concomitant, cardiovascular diseases among individuals with a diagnosis of malignancy and how this may affect the occurrence of a possible cardiomyocyte damage.

**Fig. 1:**
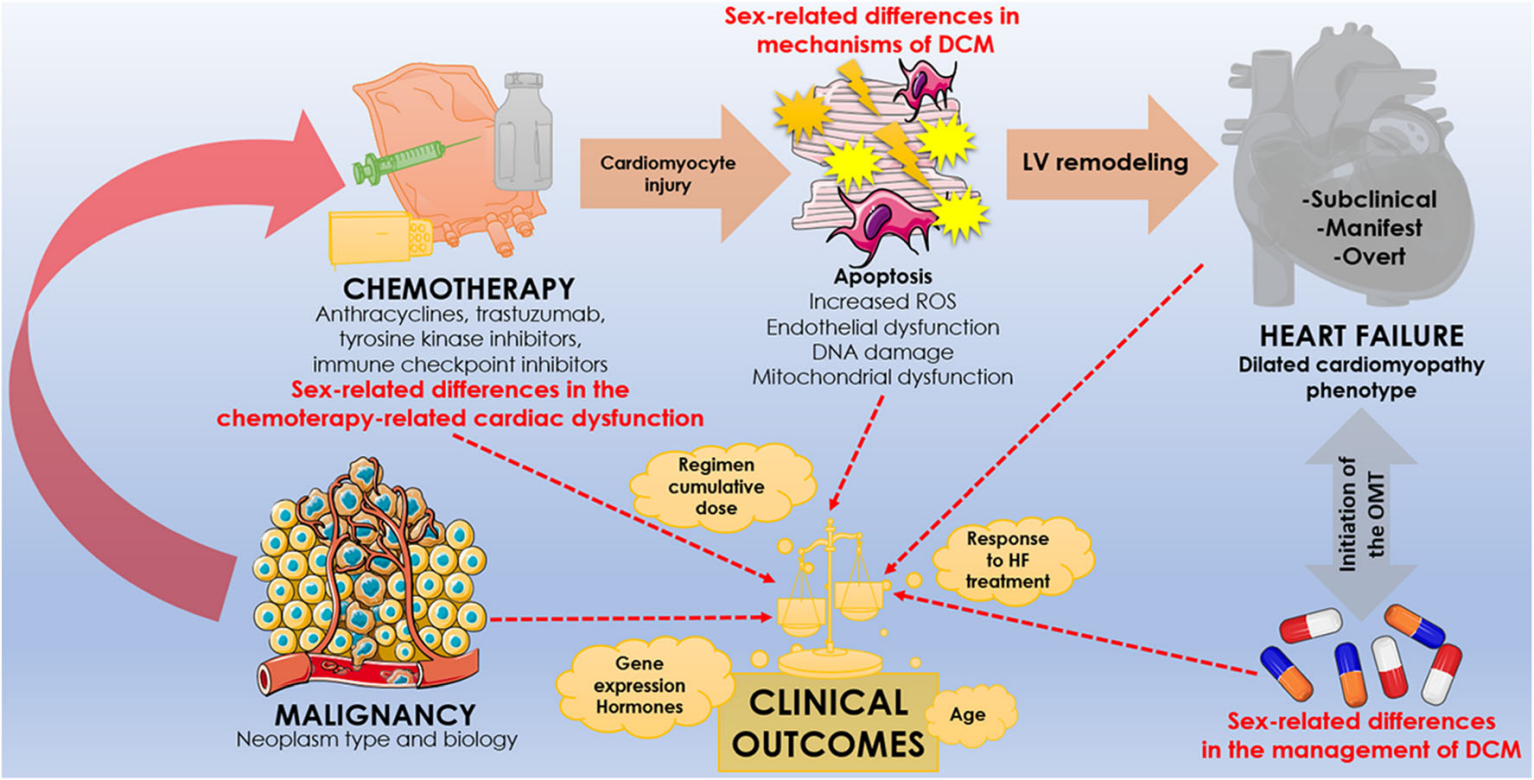
Central illustration synthesizing the main aspects of the paper. Patient-related, disease-related, and therapy-related differences induced by sex coexist, strongly impacting clinical manifestations, prognosis, and management. The integration of these aspects in clinical practice may favor the development of patient-tailored management strategies in the era of personalized medicine

This consideration is particularly appropriate when the heterogeneity of heart disease presentation between genders is evaluated. To add another level of complexity, a consideration has to be done also when describing the incidence and prevalence of cancer disease among genders and sex-specific anticancer therapies (Fig. 1).

## Sex-Related Differences in Mechanisms and Management of Dilated Cardiomyopathy

We will structure this review dividing the dissertation on DCM in three different areas:

1. Sex-related differences in mechanisms of DCM.
2. Sex-related differences in chemotherapy-related cardiac dysfunction.
3. Sex-related differences in the management of dilated cardiomyopathy.

### Sex-Related Differences in Mechanisms of DCM

Male sex is an important risk factor for developing HF following a number of cardiovascular conditions, including cardiomyopathies. However, few clinical studies have scrutinized sex differences in incidence or pathogenetic mechanisms in DCM specifically. In this regard, one study found that men with acute DCM had a higher apoptosis-related protein expression than women [29]*.* In animal models of myocarditis, the entire population of male mouse developed fibrosis and DCM after acute myocarditis, while 20% of females only developed mild DCM [30]*.*

Testosterone has been found to increase the number of M2 macrophages that express CD11b, Toll-like receptor 4, and IL-1β in animal models, leading to increased cardiac inflammation, remodeling, and DCM [31–37]. In a study of a population of patients with clinically symptomatic severe myocarditis, men were twice as likely than women to present with evidence of myocardial fibrosis [31]. Sex may contribute to the delineation of the DCM phenotype. In fact, the alteration of the functions of immune cells and platelets induced by the sex hormone-receptor interaction influences the type of cardiac inflammation, remodeling, and thrombosis involved in DCM [32, 38]. Many of the same inflammatory cells and cytokines that drive dilatation also predispose patients with DCM to thromboembolic events, including complement and platelet activation [38, 39]. In contrast, androgens have been shown to promote hypertrophy in animal models [40, 41]. It is important to underline that the activation of the estrogen receptors, mostly found in women, by 17β-estradiol prevents the apoptosis of cardiomyocyte, inhibits cardiac damage induced by reactive oxygen species, and reduces cardiac hypertrophy and fibrosis [42]*.* In a recent work by Cannatà et al., female sex resulted, in a large population of DCM patients (*n* = 1113 of total prospectively enrolled patients; *n* = 586 selected for analysis), as an independent favorable predictor for long-term cardiovascular outcomes (including cardiovascular mortality/need for heart transplantation or ventricular assist device) [43]*.*

### Sex-Related Differences in Chemotherapy-Related Cardiac Dysfunction

A specific mechanism of DCM is related to toxic effects of drugs, in particular anti-blastic drugs. Cardio-oncology experts have used for more than a decade the terms “Type I” and “Type II” cardiotoxicity in order to describe distinct patterns of cardiac dysfunction; in particular, Type I dysfunction (also termed “myocardial damage”) refers to an irreversible, dose-dependent toxicity, in which the majority of chemotherapies with cardiac involvement are included (e.g., anthracyclines and anthracycline similar compounds). On the other hand, Type II toxicity, or “myocardial dysfunction,” regards drugs, including trastuzumab, which give a transient, dose-independent damage [23••]*.* This classification may be judged archaic if we consider that LVEF recovery and cardiac event reduction in anthracycline-induced cardiotoxicity (AIC) may be achieved when cardiac dysfunction is detected early and a modern HF treatment is promptly initiated [44]. Moreover, although an initial EF assessment remains a fundamental tool to define, prognosticate, and guide treatment in HF, the implications of longitudinal EF change are becoming increasingly recognized, considering that approximately 25% of patients with HFrEF at baseline transitioned to a higher category and that 10% of patients showed complete recovery, in particular when less severe HF, fewer comorbidities, and shorter HF duration were detected [45]*.*

Early identification of cardiac damage is of paramount importance in this context, in order to avoid progression to cardiomyocyte death. At the same time, appropriate long-term follow-up allows to identify cancer survivors who are at highest risk of HF development and interrupt disease progression [46]. Many efforts have been done to identify efficient instruments to predict, diagnose, and follow cases of with cardiotoxicity; troponins (Tns) and with brain natriuretic peptide (BNP) are the two most established biomarkers in cardio-oncology. Tns (I-T) serial changes are associated with variations of EF after chemotherapy administration: Sawaya et al. found that elevated high-sensitive troponin I (TnI) levels, together with echocardiographic markers of myocardial deformation, predicted the occurrence of cardiotoxicity among breast cancer patients receiving anthracycline and trastuzumab [47]. Moreover, Ky et al. demonstrated that an early rise in high-sensitivity TnI from baseline to 3 months was associated with an increased cardiotoxicity risk among similar patients [48]. In a subgroup analysis of the HERA study, the presence of raised high-sensitive troponin before trastuzumab therapy was associated with more than twofold increased risk of subsequent LVEF deterioration [49]*.* The use of natriuretic peptides to detect HF is widely acknowledged, and even very low levels can identify high-risk patients and guide therapy, even if their role in routine surveillance is not established [23••]*.* Novel biomarkers are assuming an emerging role in detecting acute myocardial damage by anti-blastic drugs; in particular, soluble ST2 (sST2), miR-126-3p, miR-199a-3p, miR-423-5p, and miR-34a-5p may represent as innovative biomarkers for potential early and sensitive detection of the cardiomyopathy associated with anthracycline-based breast cancer chemotherapy [47]*.* It must be defined whether emerging biomarkers in acute or chronic HF may have a position in cardiotoxicity [50]*.*

The effects on cardiomyocytes mediated by these drugs are at the same time conditioned by sex hormones and sex-related gene expression. These differences result from the presence of women specific forms of primary disease and sex-specific biological response. The most common forms of CRCD are secondary to breast cancer treatment, which is strongly sex-oriented, followed by hematological malignancies [22, 51]. Women with CRCD that progressed to DCM are older, have smaller end-diastolic volume index, have higher left ventricular EF, have a lower burden of scar at CMR, have a worse NYHA class but a better long-term prognosis, and have higher rate of previous chemotherapy exposure compared with women with the diagnosis of primary DCM [52–54]. Below, we will focus on main sex-specific characteristics of CRCD with a brief review of biological mechanisms possibly involved according to the specific chemotherapy regimen used. Principal studies evaluating sex differences in anti-blastic drugs’ cardiac effects are exposed in Table 2.

**Table 2:** Principal studies which evaluated sex-related chemotherapy-induced cardiotoxicity

Study	Patients (F/M)	Time after therapy (year)	Sex-related differences
Anthracycline			
Increased female risk			
Silber et al. 1993 [55]	65/85	4.7	Female were at higher risk of cardiac dysfunction than males; the odds ratio for having an abnormal angiographic and electrocardiographic test result was higher for females than for males.
Lipshultz et al. 1995 [56]	40/47	2	Female sex and a higher cumulative dose of DOX were associated with depressed contractility, and there was an interaction between these two variables.
Increased male risk			
Myrehaug et al. 2010 [57]	480/616	10	Male sex was a significant risk factor for cardiac hospitalization among Hodgkin lymphoma patients.
Meiners et al. 2018 [58]	615	15	For females and males treated with DOX plus mediastinal radiation therapy, the estimated 15-year incidence rate of cardiac hospitalization was higher in male.
Tyrosine kinase receptor inhibitors			
Increased female risk			
Van der Veldt et al. 2008 [59]	27/55	1	Female sex was significantly related with severe toxicity after sunitinib treatment (*p* = 0.006).
Bowles et al. 2012 [60]	12,500	nd	Anthracycline and trastuzumab were associated with increased HF and cardiomyopathy risk compared with no chemotherapy in an exclusively female study population.
Immune checkpoint inhibitors			
Increased male benefit			
Conforti et al. 2018 [61]	3705/7646	n/d	Men benefit more from ICI treatment than women.
Chitturi et al. 2019 [62]	102/140	0.5	No differences according to sex in terms of incidence of complication and MACE occurrence.

*DOX* doxorubicin, *HF* heart failure, *MACE* major adverse cardiovascular events

#### Anthracycline-Induced Cardiotoxicity: Available Evidences and Controversies

One of the most common classes of chemotherapeutic agents, the anthracyclines, is employed in the treatment of a wide variety of solid organ tumors and hematologic malignancies, including leukemia, lymphoma, breast cancer, lung cancer, multiple myeloma, and sarcoma [24]. Anthracyclines inhibit DNA/RNA synthesis by intercalating between base pairs of the DNA/RNA strand [24]. It is well known that anthracyclines are responsible for a Type I cardiotoxicity, characterized by cardiomyocyte death, either through necrosis or apoptosis, resulting in an irreversible damage [24].

The impact of sex in determination and progression of related cell death is poorly understood. In a large cohort of patients treated with anthracyclines for different types of malignancies, major adverse cardiac events (symptomatic heart failure or cardiac death) were more frequent in men than in women [63]. However, in a study with 150 patients treated with the same drugs (65 girls and 85 boys), Silber et al. reported that women were at higher risk of cardiac dysfunction than men, with the odds ratio for having an abnormal gated nuclear angiography and electrocardiogram result being 3.2 for females versus males [55].

Significant sex-related differences were observed in a study with a cohort of 120 patients treated with doxorubicin (DOX). In particular, an increase in LV size and decrease in LV mass were predominant in female subjects, concluding that the female sex was an independent risk factor for cardiac abnormalities after treatment with DOX in cancer [56••]. In a study of Myrehaug et al., male sex was a significant risk factor for cardiac hospitalization among Hodgkin lymphoma patients [57, 58]. The lack of consistency in study designs and the different definitions of cardiotoxicity preclude reaching consensus regarding the role of sex as a risk factor in AIC, even though the scientific community agrees with considering female sex a risk factor for AIC, in the same manner as advanced age, prior mediastinal radiation therapy, hypertension, concomitant treatment with cyclophosphamide, trastuzumab or paclitaxel, and prior cardiac disease [26].

#### Targeted Therapies: Tyrosine Kinase Receptor Inhibitors

Receptor tyrosine kinases (RTKs) are the high-affinity cell surface receptors for many polypeptide growth factors, cytokines, and hormones that have been shown not only to be key regulators of normal cellular processes but also to have a critical role in the development and progression of many types of cancer. Trastuzumab is a recombinant humanized monoclonal antibody that inhibits tyrosine kinase receptor expressed on cancer cells; the same enzyme is also expressed on cardiomyocytes and its inhibition seldom provokes DCM, in a mostly reversible and dose-independent fashion (Type II cardiotoxicity) [64]. At the one hand, the most severe complication of trastuzumab therapy involves its potential to adversely affect cardiac function; however, the exact mechanism of this toxicity remains unclear [64]. The risk of trastuzumab-related cardiac events increases when additional cardiovascular disease risk factors are noted, especially a history of coronary artery disease (CAD) or impaired left ventricular dysfunction [65], and when trastuzumab belongs to a combined scheme with anthracyclines. Nevertheless, trastuzumab use is predominantly confined to breast cancer management, with little application in gastric neoplasms; for this reason, no data are available on sex differences in cardiovascular toxicity.

Tyrosine kinase inhibitors (TKIs) are a novel class of anticancer drugs for which the sexual dimorphism has also been described. The use of these drugs is limited by their cardiotoxicity [51]. They are small molecules that occupy the ATP binding site of the tyrosine kinase receptor inhibiting the abnormal high kinase activity and uncontrolled cell growth [64]. Sunitinib belongs to TKIs exhibiting cardiotoxicity. A recent study highlights sex difference in sunitinib-related cardiotoxicity; in this case, female appear more sensitive than males to the toxicity of sunitinib, showing more susceptibility to multi-organ system toxicity [59]. After all, toxicities related to imatinib and sorafenib appear not to be sex-associated, as far as drug efficacy [66].

#### Targeted Therapies: Immune Checkpoint Inhibitors

The mechanisms of the most commonly used immune checkpoint inhibitors (ICIs) are based on blocking either the cytotoxic T-lymphocytes-associated antigen-4 (CTLA-4) or programmed cell death protein 1 (PD-1) pathways. Cardiac immune-related adverse events appear both in mono- and combination therapy. The exact mechanism of cardiac immune-related adverse effects remains poorly understood; however, it is likely related to the direct inhibition of PD-1 and CTLA-4. Increased expression of PD-1 has been reported on cardiomyocytes from rat hearts that underwent ischemia reperfusion. A recent study suggests that male patients obtain more benefits from ICIs vs control compared with female patients [66]. Conforti et al. suggest that ICIs can improve overall survival for patients with advanced cancers such as melanoma and non-small-cell lung cancer and magnitude of benefit is sex-dependent. This meta-analysis highlights a significant difference in the efficacy of ICIs between men and women, when compared with controls for each sex. The pooled reduction of risk of death was double the size for male patients than for female patients [61]. A recent work by Chitturi et al., investigating cardiac adverse events in patients treated with ICIs for lung tumors, showed no dissimilarities, according to sex in terms of incidence of complications and major adverse cardiac events (MACE) occurrence. Given the recent approval of this class of drugs, only limited data are available about cardiac toxicity as well as about sex differences.

### Sex-Related Differences in the Management of DCM

In the era of precision medicine, the sex and gender differences are still neglected in clinical research. Notably, a recent systematic review of all randomized control trials pointed out that women are still less enrolled in clinical trials and the involvement of gender is even less studied in comparison with sex [67]*.*

Because women are underrepresented in clinical trials, current guidelines do not recommend different sex-related therapies for HF, but not all therapies may be adequate due to differences in response to drugs. Differences in response to drugs might be due to different bioavailability, sex-specific amount and distribution of body fat, different metabolisms, and renal clearance [68]. Principal sex differences highlighted by clinical trials investigating the effects of DCM drugs are exposed in Table 3.

**Table 3:** Differences in female sex enrollment in main clinical trials evaluating the pharmacological therapies for chronic heart failure (CHF)

Study	Drug	Mean LVEF (%)	Number of women	Total pts. enrolled	Women (%)
BBs					
MERIT-HF [69]	Metoprolol CR/XL	28	458	1990	23
COPERNICUS [70]	Carvedilol	20	412	2289	18
CIBIS-II [71]	Bisoprolol	28	514	2539	20
SENIORS [72]	Nebivolol	33	785	2128	37
ACEi/ARBs					
CHARM [73]	Candesartan	31	925	3855	24
Val-HEFT [74]	Valsartan	27	1002	5010	20
CONSENSUS [75]	Enalapril	Unknown	59	253	23
SOLVD [76]	Enalapril	25	514	2569	20
MRAs					
RALES [77]	Spironolactone	25	446	1663	27
EPHESUS [78]	Eplerenone	26	610	2737	22
I_f_-channel blocker					
J-SHIFT [79]	Ivabradine	29	1561	6505	24
Digoxin					
DIG [80]	Digoxin	28	1496	6800	22
ARNi					
PARADIGM-HF [16]	Sacubitril-valsartan	< 35	1832	8399	22
PIONEER-HF [81]	Sacubitril-valsartan	24	246	881	28

*ACEi* angiotensin-converting enzyme inhibitors, *ARB* angiotensin receptor blockers, *ARNi* angiotensin receptor-neprilysin inhibitors, *BB* beta-blockers, *CHARM* Candesartan in Heart failure: Assessment of Reduction in Mortality and Morbidity, *CIBIS-II* Cardiac Insufficiency Bisoprolol Study II, *CONSENSUS* Co-operative North Scandinavian Enalapril Survival Study, *COPERNICUS* Carvedilol Prospective Randomized Cumulative Survival, *DIG* Digitalis Investigation Group, *EPHESUS* Eplerenone Post-Acute Myocardial Infarction Heart Failure Efficacy and Survival Study, *J-SHIFT* Japanese Patients With Chronic Heart Failure, *MERIT-HF* Metoprolol CR/XL Randomized Intervention Trial in-Congestive Heart Failure, *MRA* mineralocorticoid-receptor antagonists, *PARADIGM-HF* Prospective Comparison of ARNI With ACEI to Determine Impact on Global Mortality and Morbidity in Heart Failure trial, *PIONEER-HF* Comparison of Sacubitril/Valsartan Versus Enalapril on Effect on NT-pro BNP in Patients Stabilized From an Acute Heart Failure Episode, *RALES* Randomized Aldactone Evaluation Study, *SENIORS* randomized trial to determine the effect of nebivolol on mortality and cardiovascular hospital admission in elderly patients with heart failure, *SOLVD* Studies of Left Ventricular Dysfunction, *Val-HeFT* Valsartan Heart Failure Trial

Inhibitors of the renin angiotensin aldosterone system (RAAS) have been shown to improve the outcome of HFrEF in a large number of controlled randomized trials. Angiotensin-converting enzyme (ACE) inhibitors are a cornerstone of HF management and have been found to reduce morbidity and mortality in HFrEF patients [68]*.* This drug category finds indication in all symptomatic patients with EF < 35% [68]. Early landmark trials with ACE inhibitors suggested that reductions in mortality and HF hospitalizations were observed in men but not in women with HFrEF. However, the Studies of Left Ventricular Dysfunction (SOLVD), which enrolled a small percentage of women, showed a reduction in combined mortality or HF hospitalization from 39.5% in the placebo arm to 29.7% in the ACE inhibitor enalapril arm in men compared with non-significant reduction from 38.7 to 37% in women [76].

If ACE inhibitors are contraindicated or not tolerated, angiotensin II receptor blockers (ARBs) should be used, with similar efficacy [68]. Trials testing ARBs in HF have achieved similar benefit on survival and HF hospitalization in men and women with HFrEF. In the CHARM (Candesartan in Heart failure Assessment of Reduction in Mortality and morbidity) trial, the benefit of candesartan regarding reduction of all-cause mortality and HF hospitalization in patients with systolic HF was similar in women and men [73].

Beta-blockers are complementary to ACE inhibitors in the treatment of symptomatic HFrEF, giving an additional advantage in terms of morbidity and mortality, even though not recommended in decompensated and acute states [68]. Pooled mortality data by sex from the MERIT-HF (Metoprolol Cr/Xl Randomized Intervention Trial in Congestive Heart Failure) [69], CIBIS (Cardiac Insufficiency Bisoprolol Study)-II [71], and COPERNICUS (Effect of Carvedilol on Survival in Severe Chronic Heart Failure) [70] trials showed similar and significant survival benefits in women and men.

Mineralocorticoid/aldosterone receptor antagonists (MRAs) block receptors that bind aldosterone and, with different degrees of affinity, other steroid hormone (e.g., corticosteroids, androgens) receptors [68]. Spironolactone or eplerenone is recommended in all symptomatic patients (despite treatment with an ACEI and a beta-blocker) with HFrEF and LVEF ≤ 35%, to reduce mortality and HF hospitalization. In the Randomized ALdactone Evaluation Study (RALES) trial [77] and in the Eplerenone Post-myocardial infarction Heart failure Efficacy and Survival Study (EPHESUS) trial [78], no sex differences in prognosis were noted, although women represented only the 27% and 28% of patients, respectively.

A recently introduced compound, sacubitril-valsartan (SV) (LCZ696), combines the moieties of an ARB and a neprilysin inhibitor (sacubitril) and has recently been shown to be superior to an ACE inhibitor (enalapril) in reducing the risk of death and of hospitalization for HF, both in the acute and chronic setting [68, 82]. A recent retrospective study by Vincent et al. investigated sex-specific differences in efficacy, tolerability, and safety of SV in real-world heart failure with reduced HFrEF patients [83]*.* SV in women has a similar tolerability as in men and females seem to have a more frequent functional class improvement than males [83]*.* Although no sex-related meaningful differences have been reported in SV pharmacokinetics and pharmacodynamics [82, 84], a more sex-balanced recruitment would be desirable. At the same time, dedicated clinical trials in patients with CRCD and HFrEF are lacking and these cases follow recommendations expressed by current guidelines.

Digoxin primarily inhibits the sodium potassium adenosine triphosphatase channel in particular at myocardium level, with well-known inotropic and anti-arrhythmic effects [85]. Digoxin is no longer the first choice for the treatment of HFrEF, even though it may be considered in symptomatic HF patients with sinus rhythm despite treatment with an ACE inhibitor (or ARB), a beta-blocker, and an MRA, to reduce the risk of hospitalization, or in patients with high-rate atrial fibrillation [68]. Yet, a post hoc sub-analysis of the Digitalis trial showed that digoxin therapy was associated with an increased risk of death from any cause among women, but not among men, with HF and depressed left ventricular systolic function [86•]. Furthermore, findings from a prospective multinational database from Europe, and validating results in a multinational population from Asia, suggest that no additional benefit may be gained as the dose of guideline-directed HFrEF medications is up-titrated in women and importantly draw attention to the potential need for different sex-based dose targets in HFrEF [87].

## Conclusion: Perspectives in Personalized Gender Medicine—the Expert Point of View

DCM show many sex-related differences in prevalence, etiology, phenotype expression, response to therapy, and outcomes. In particular, in CRCD, the underlying pathophysiological mechanisms, although extensively investigated, are poorly understood also because of the continuous evolution in drug development. Moreover, cancer population has never been included in pivotal clinical studies and so, the adequate evaluation of sex differences in drug response is missing. A better understanding of these mechanisms could lead to the definition of a patient-tailored management strategy.

Cardio-oncology is a medical subspecialty dedicated to providing comprehensive CV care to cancer patients from cancer diagnosis to survivorship. The primary focus is to support cancer patients through CV risk stratification and CV monitoring during and after cancer treatments, as well as treatment of pre-existing and newly diagnosed CV disease. The key element for high-risk patients is preventive measures depending on the associated risk factors in order to preserve CV health. In those with heart disease, a multidisciplinary approach to cancer therapy choice would be most appropriate to minimize cardiotoxicity. Prevention and management strategies of cardiotoxicity will be important to allow for optimal cancer therapy while protecting CV health, and thus to improve both for cardiological and oncological outcomes.

## References

[1] McKenna WJ, Maron BJ, Thiene G (2017). Classification, epidemiology, and global burden of cardiomyopathies. Circ Res.

[2] Maron BJ, Towbin JA, Thiene G, Antzelevitch C, Corrado D, Arnett D, Moss AJ, Seidman CE, Young JB; American Heart Association; Council on Clinical Cardiology, Heart Failure and Transplantation Committee; Quality of Care and Outcomes Research and Functional Genomics and Translational Biology Interdisciplinary Working Groups; Council on Epidemiology and Prevention Circulation 2006; 113:1807–16. 10.1161/CIRCULATIONAHA.106.174287 16567565

[3] Elliott P, Andersson B, Arbustini E, Bilinska Z, Cecchi F, Charron P, et al. Classification of the cardiomyopathies: a position statement from the European Society of Cardiology Working Group on Myocardial and Pericardial Diseases. Eur Heart J. 2008;29:270–6. 10.1093/eurheartj/ehm342 17916581

[4] Hershberger RE, Hedges DJ, Morales A (2013). Dilated cardiomyopathy: the complexity of a diverse genetic architecture. Nat Rev Cardiol.

[5] Hershberger RE, Morales A, Siegfried JD (2010). Clinical and genetic issues in dilated cardiomyopathy: a review for genetics professionals. Genet Med.

[6] Richardson P, McKenna W, Bristow M, Maisch B, Mautner B, O’Connell J, et al. Report of the 1995 World Health Organization/International Society and Federation of Cardiology Task Force on the definition and classification of cardiomyopathies. Circulation. 1996;96:841–2. 10.1161/01.cir.93.5.841 8598070

[7] Bloomfield GS, Alenezi F, Barasa FA, Lumsden R, Mayosi BM, Velazquez EJ (2015). Human immunodeficiency virus and heart failure in low- and middle-income countries. JACC: Heart Failure.

[8] Pereira Nunes MC, Beaton A, Acquatella H, Bern C, Bolger AF, Echeverría LE, et al. Chagas cardiomyopathy: an update of current clinical knowledge and management, a scientific statement from the American Heart Association. Circulation. 2018;138:169–209. 10.1161/CIR.0000000000000599 30354432

[9] Sinagra G, Fabris E, Romani S, Negri F, Stolfo D, Brun F, Merlo M. Dilated cardiomyopathy at the crossroad: multidisciplinary approach. 2019 May 18. In: Sinagra G, Merlo M, Pinamonti B, editors. Dilated cardiomyopathy: from genetics to clinical management. Cham (CH): Springer; 2019. Chapter 15. 32091714

[10] Feingold B, Mahle WT, Auerbach S, Clemens P, Domenighetti AA, Jefferies JL, et al. Management of cardiac involvement associated with neuromuscular diseases: a scientific statement from the American Heart Association. Circulation. 2017;136:200–31. 10.1161/CIR.0000000000000526 28838934

[11] Stergiopoulos K, Lima FV (2019). Peripartum cardiomyopathy-diagnosis, management, and long term implications. Trends Cardiovasc Med.

[12] Hantson P (2019). Mechanisms of toxic cardiomyopathy. Clin Toxicol (Phila).

[13] Towbin JA, Lowe AM, Colan SD, Sleeper L, Orav EJ, Clunie S, et al. Incidence, causes, and outcomes of dilated cardiomyopathy in children. JAMA. 2006;296:1867–76. 10.1001/jama.296.15.1867 17047217

[14] Vos T (2016). (GBD 2015 Disease and Injury Incidence and Prevalence Collaborators). Global, regional, and national incidence, prevalence, and years lived with disability for 310 diseases and injuries, 1990–2015: a systematic analysis for the Global Burden of Disease Study 2015. Lancet.

[15] Lozano R, Naghavi M, Foreman K, Lim S, Shibuya K, Aboyans V, et al. Global and regional mortality from 235 causes of death for 20 age groups in 1990 and 2010: a systematic analysis for the Global Burden of Disease Study 2010. Lancet. 2012;380:2095–128. 10.1016/S0140-6736(12)61728-0 PMC10790329 23245604

[16] Balmforth C, Simpson J, Shen L, Jhund PS, Lefkowitz M, Rizkala AR, et al. Outcomes and effect of treatment according to etiology in HFrEF: an analysis of PARADIGM-HF. JACC Heart Fail. 2019;7:457–65. 10.1016/j.jchf.2019.02.015 31078482

[17] Saxon LA, Stevenson WG, Middlekauff HR, Fonarow G, Woo M, Moser D, et al. Predicting death from progressive heart failure secondary to ischemic or idiopathic dilated cardiomyopathy. Am J Cardiol. 1993;72:62–5. 10.1016/0002-9149(93)90220-7 8517430

[18] Bozkurt B, Colvin M, Cook J, Cooper LT, Deswal A, Fonarow GC, et al. Current diagnostic and treatment strategies for specific dilated cardiomyopathies: a scientific statement from the American Heart Association. Circulation. 2016;134:579–646. 10.1161/CIR.0000000000000455 27832612

[19] Dries DL, Exner DV, Gersh BJ, Cooper HA, Carson PE, Domanski MJ (1999). Racial differences in the outcome of left ventricular dysfunction. N Engl J Med.

[20] Fairweather D, Cooper LT, Blauwet LA (2013). Sex and gender differences in myocarditis and dilated cardiomyopathy. Curr Probl Cardiol.

[21] Dagenais GR, Leong DP, Rangarajan S, Lanas F, Lopez-Jaramillo P, Gupta R, et al. Lancet. 2020;395:785–94. 10.1016/S0140-6736(19)32007-0 31492501

[22] Araujo-Gutierrez R, Ibarra-Cortez SH, Estep JD, Bhimaraj A, Guha A, Hussain I, et al. Incidence and outcomes of cancer treatment-related cardiomyopathy among referrals for advanced heart failure. Cardiooncology. 2018;4:3. 10.1186/s40959-018-0029-y. 10.1186/s40959-018-0029-y PMC7048122 32154004

[23] •• Zamorano JL, Lancellotti P, Muñoz DR, Aboyans V, Asteggiano R, Galderisi M, et al. ESC Position Paper on cancer treatments and cardiovascular toxicity developed under the auspices of the ESC Committee for Practice Guidelines: The Task Force for cancer treatments and cardiovascular toxicity of the European Society of Cardiology (ESC). Eur Heart J. 2016;37:2768–801. 10.1093/eurheartj/ehw211**Paper of paramount importance in definition, imaging, and management of cardiac complications in chemotherapy.** 10.1093/eurheartj/ehw211 27567406

[24] Perez IE, Taveras Alam S, Hernandez GA, Sancassani R (2019). Cancer therapy-related cardiac dysfunction: an overview for the clinician. Clin Med Insights Cardiol.

[25] Oliveira GH, Dupont M, Naftel D, Myers SL, Yuan Y, Tang WH, et al. Increased need for right ventricular support in patients with chemotherapy-induced cardiomyopathy undergoing mechanical circulatory support: outcomes from the INTERMACS Registry (Interagency Registry for Mechanically Assisted Circulatory Support). J Am Coll Cardiol. 2014;63:240–8. 10.1016/j.jacc.2013.09.040 24161324

[26] Yeh ET, Bickford CL (2009). Cardiovascular complications of cancer therapy: incidence, pathogenesis, diagnosis, and management. J Am Coll Cardiol.

[27] Oliveira GH, Hardaway BW, Kucheryavaya AY, Stehlik J, Edwards LB, Taylor DO (2012). Characteristics and survival of patients with chemotherapy-induced cardiomyopathy undergoing heart transplantation. J Heart Lung Transplant.

[28] Friedrich EB, Böhm M (2007). Management of end stage heart failure. Heart..

[29] Sheppard R, Bedi M, Kubota T, Semigran MJ, Dec W, Holubkov R, et al. Myocardial expression of fas and recovery of left ventricular function in patients with recent-onset cardiomyopathy. J Am Coll Cardiol. 2005;46:1036–42. 10.1016/j.jacc.2005.05.067 16168288

[30] Coronado MJ (2012). Testosterone and interleukin-1β increase cardiac remodeling during coxsackievirus B3 myocarditis via serpin A 3n. Am J Physiol Heart Circ Physiol.

[31] Cocker MS, Abdel-Aty H, Strohm O, Friedrich MG (2009). Age and gender effects on the extent of myocardial involvement in acute myocarditis: a cardiovascular magnetic resonance study. Heart..

[32] Schultheiss H, Fairweather D, Caforio ALP (2019). Dilated cardiomyopathy. Nat Rev Dis Primers.

[33] Baldeviano GC, Barin JG, Talor MV, Srinivasan S, Bedja D, Zheng D, et al. Interleukin-17A is dispensable for myocarditis but essential for the progression to dilated cardiomyopathy. Circ Res. 2010;106:1646–55. 10.1161/CIRCRESAHA.109.213157 20378858

[34] Myers JM (2016). Cardiac myosin-Th17 responses promote heart failure in human myocarditis. JCI Insight.

[35] Diny NL, Baldeviano GC, Talor MV, Barin JG, Ong SF, Bedja D, et al. Eosinophil-derived IL-4 drives progression of myocarditis to inflammatory dilated cardiomyopathy. J Exp Med. 2017;214:943–57. 10.1084/jem.20161702 PMC5379983 28302646

[36] Frisancho-Kiss S (2009). Gonadectomy of male BALB/c mice increases Tim-3(+) alternatively activated M2 macrophages, Tim-3(+) T cells, Th2 cells and Treg in the heart during acute coxsackievirus-induced myocarditis. Brain Behav. Immun.

[37] Fairweather D, Coronado MJ, Garton AE, Dziedzic JL, Bucek A, Cooper LT Jr, et al. Sex differences in translocator protein 18 kDa (TSPO) in the heart: implications for imaging myocardial inflammation. J Cardiovasc Transl Res. 2014;7:192–202. 10.1007/s12265-013-9538-0 PMC3951973 24402571

[38] Regitz-Zagrosek V, Kararigas G (2017). Mechanistic pathways of sex differences in cardiovascular disease. Physiol Rev.

[39] Abston ED, Barin JG, Cihakova D, Bucek A, Coronado MJ, Brandt JE, et al. IL-33 independently induces eosinophilic pericarditis and cardiac dilation: ST2 improves cardiac function. Circ Heart Fail. 2012;5:366–75. 10.1161/CIRCHEARTFAILURE.111.963769 PMC3874395 22454393

[40] Melchert RB, Welder AA (1995). Cardiovascular effects of androgenic-anabolic steroids. Med Sci Sports Exerc.

[41] Scheuer J, Malhotra A, Schaible TF, Capasso J (1987). Effects of gonadectomy and hormonal replacement on rat hearts. Circ Res.

[42] Vitale C, Mendelsohn ME, Rosano GMC (2009). Gender differences in the cardiovascular effect of sex hormones. Nat Rev Cardiol.

[43] Cannatà A, Fabris E, Merlo M, Artico M, Gentile P, Pio Loco C, Ballaben M, Ramani F, Barbati G, Sinagra G (2020). Sex differences in the long-term prognosis of dilated cardiomyopathy. Can Journal of Cardiology.

[44] Cardinale D, Colombo A, Lamantia G, Colombo N, Civelli M, De Giacomi G, Rubino M, Veglia F, Fiorentini C, Cipolla CM (2010). Anthracycline-induced cardiomyopathy: clinical relevance and response to pharmacologic therapy. J Am Coll Cardiol.

[45] Savarese G, Vedin O, D’Amario D, Uijl A, Dahlström U, Rosano G, Lam CSP, Lund LH (2019). Prevalence and prognostic implications of longitudinal ejection fraction change in heart failure. JACC Heart Fail..

[46] Camilli M, Del Buono MG, Crea F, Minotti G (2020). Acute heart failure twenty-nine years after treatment for childhood cancer. JACC:Cardio Oncology.

[47] Frères P, Bouznad N, Servais L, Josse C, Wenric S, Poncin A, Thiry J, Moonen M, Oury C, Lancellotti P, Bours V, Jerusalem G (2018). Variations of circulating cardiac biomarkers during and after anthracycline-containing chemotherapy in breast cancer patients. BMC Cancer.

[48] Ky B, Putt M, Sawaya H, French B, Januzzi JL, Sebag IA, Plana JC, Cohen V, Banchs J, Carver JR, Wiegers SE, Martin RP, Picard MH, Gerszten RE, Halpern EF, Passeri J, Kuter I, Scherrer-Crosbie M (2014). Early increases in multiple biomarkers predict subsequent cardiotoxicity in patients with breast cancer treated with doxorubicin, taxanes, and trastuzumab. J Am Coll Cardiol.

[49] Zardavas D, Suter TM, Van Veldhuisen DJ, Steinseifer J, Noe J, Lauer S (2017). Role of troponins I and T and N- terminal prohormone of brain natriuretic peptide in monitoring cardiac safety of patients with early-stage human epidermal growth factor receptor 2-positive breast cancer receiving trastuzumab: a Herceptin adjuvant study cardiac marker substudy. J Clin Oncol.

[50] Borovac JA, Glavas D, Grabovac ZS, Domic DS, D’Amario D, Bozic J (2019). Catestatin in acutely decompensated heart failure patients: insights from the CATSTAT-HF study. J Clin Med.

[51] Howard E, Steingart RM, Armstrong GT, Lyon AR, Armenian SH, Teresa Voso M, Cicconi L, Coco FL, Minotti G (2019). Cardiovascular events in cancer survivors. Semin Oncol.

[52] Vary TC, Kimball SR, Sumner A (2007). Sex-dependent differences in the regulation of myocardial protein synthesis following long-term ethanol consumption. Am J Physiol Regul Integr Comp Physiol.

[53] Vitali Serdoz L, Lutman C, Cadamuro E, Barbati G, Zecchin M, Merlo M, Puggia I, Pinamonti B, Di Lenarda A, Sinagra G (2017). Conflicting gender-related differences in the natural history of patients with idiopathic dilated cardiomyopathy. Epidemiol Biostat Public Health.

[54] Halliday BP, Gulati A, Ali A, Newsome S, Lota A, Tayal U, Vassiliou VS, Arzanauskaite M, Izgi C, Krishnathasan K, Singhal A, Chiew K, Gregson J, Frenneaux MP, Cook SA, Pennell DJ, Collins P, Cleland JGF, Prasad SK (2018). Sex- and age-based differences in the natural history and outcome of dilated cardiomyopathy. Eur J Heart Fail.

[55] Silber JH, Jakacki RI, Larsen RL, Goldwein JW (1993). Barber G increased risk of cardiac dysfunction after anthracyclines in girls. Med Pediatr Oncol.

[56••] Lipshultz SE, Lipsitz SR, Mone SM, Goorin AM, Sallan SE, Sanders SP, et al. Female sex and higher drug dose as risk factors for late cardiotoxic effects of doxorubicin therapy for childhood cancer. N Engl J Med. 1995;332:1738–43. **The article provide one of the first and most comprehensive description of prospective changes of echocardiographic variables in patients exposed to doxorubicin, highlighting the importance of sex for risk stratification**. 10.1056/NEJM199506293322602 7760889

[57] Myrehaug S, Pintilie M, Yun L, Crump M, Tsang RW, Meyer RM, Sussman J, Yu E, Hodgson DC (2010). A population-based study of cardiac morbidity among Hodgkin lymphoma patients with preexisting heart disease. Blood..

[58] Meiners B, Shenoy C, Zordoky BN (2018). Clinical and preclinical evidence of sex-related differences in anthracycline-induced cardiotoxicity. Biol Sex Differ.

[59] Van der Veldt AA (2008). Predictive factors for severe toxicity of sunitinib in unselected patients with advanced renal cell cancer. Br J Cancer.

[60] Bowles EJ, Wellman R, Feigelson HS (2012). Risk of heart failure in breast cancer patients after anthracycline and trastuzumab treatment: a retrospective cohort study. J Natl Cancer Inst.

[61] Conforti F, Pala L, Bagnardi V, de Pas T, Martinetti M, Viale G, Gelber RD, Goldhirsch A (2018). Cancer immunotherapy efficacy and patients’ sex: a systematic review and meta-analysis. Lancet.

[62] Chitturi KR, Xu J, Araujo-Gutierrez R, Bhimaraj A, Guha A, Hussain I, Kassi M, Bernicker EH, Trachtenberg BH (2019). Immune checkpoint inhibitor-related adverse cardiovascular events in patients with lung cancer. J Am Coll Cardiol CardioOnc.

[63] Wang L, Tan TC, Halpern EF, Neilan TG, Francis SA, Picard MH, Fei H, Hochberg EP, Abramson JS, Weyman AE, Kuter I, Scherrer-Crosbie M (2015). Major cardiac events and the value of echocardiographic evaluation in patients receiving anthracycline-based chemotherapy. Am J Cardiol.

[64] Force T, Krause DS, Van Etten RA (2007). Molecular mechanisms of cardiotoxicity of tyrosine kinase inhibition. Nat Rev Cancer.

[65] Senkus E, Jassem J (2011). Cardiovascular effects of systemic cancer treatment. Cancer Treat Rev.

[66] Wang S (2019). Sex differences in cancer immunotherapy efficacy, biomarkers and therapeutic strategy. Molecules..

[67] De Bellis A, De Angelis G, Fabris E, Cannatà A, Merlo M, Sinagra G (2020). Gender-related differences in heart failure: beyond the “one-size-fits-all” paradigm. Heart Fail Rev.

[68] Ponikowski P, Voors AV, Anker SD, ESC Scientific Document Group (2016). ESC guidelines for the diagnosis and treatment of acute and chronic heart failure: the Task Force for the diagnosis and treatment of acute and chronic heart failure of the European Society of Cardiology (ESC). Eur Heart J.

[69] MERIT-HF Study Group (1999). Effect of metoprolol CR/XL in chronic heart failure: Metoprolol CR/XL Randomised Intervention Trial in-Congestive Heart Failure (MERIT-HF). Lancet..

[70] Krum H, Mohacsi P, Katus HA, Tendera M, Rouleau JL, Fowler MB, Coats AJ, Roecker EB, Packer M, Carvedilol Prospective Randomized Cumulative Survival (COPERNICUS) Study Group (2006). Are beta-blockers needed in patients receiving spironolactone for severe chronic heart failure? An analysis of the COPERNICUS study. Am Heart J..

[71] Mulder BA, Damman K, Van Veldhuisen DJ, Van Gelder IC, Rienstra M (2017). Heart rate and outcome in heart failure with reduced ejection fraction: differences between atrial fibrillation and sinus rhythm-a CIBIS II analysis. Clin Cardiol.

[72] Flather MD, Shibata MC, Coats AJ, Van Veldhuisen DJ, Parkhomenko A, Borbola J, et al. Randomized trial to determine the effect of nebivolol on mortality and cardiovascular hospital admission in elderly patients with heart failure (SENIORS). Eur Heart J. 2005;26:215–25. 10.1093/eurheartj/ehi115 15642700

[73] O’Meara E, Clayton T, McEntegart MB, McMurray JJ, Piña IL, Granger CB, Ostergren J, Michelson EL, Solomon SD, Pocock S, Yusuf S, Swedberg K, Pfeffer MA, CHARM Investigators (2007). Sex differences in clinical characteristics and prognosis in a broad spectrum of patients with heart failure: results of the Candesartan in Heart failure: Assessment of Reduction in Mortality and morbidity (CHARM) program. Circulation..

[74] Butler J, Anand IS, Kuskowski MA, Rector T, Carson P, Cohn JN, et al. Digoxin use and heart failure outcomes: results from the Valsartan Heart Failure Trial (Val-HeFT). Congest Heart Fail. 2010;16:191–5. 10.1111/j.1751-7133.2010.00161.x 20887614

[75] McMurray JJ. CONSENSUS to EMPHASIS: the overwhelming evidence which makes blockade of the renin-angiotensin- aldosterone system the cornerstone of therapy for systolic heart failure. Eur J Heart Fail. 2011;13:929–36. 10.1093/eurjhf/hfr093 21816763

[76] Limacher MC, Yusuf S, Wenger NK, Speroff L, Packard B, for the SOLVD Investigators (1993). Gender differences in presentation, morbidity and mortality in the Studies of Left Ventricular Dysfunction (SOLVD): a preliminary report. Cardiovascular health and disease in women.

[77] Pitt B, Zannad F, Remme WJ, Cody R, Castaigne A, Perez A, Palensky J, Wittes J (1999). The effect of spironolactone on morbidity and mortality in patients with severe heart failure. N Engl J Med.

[78] Pitt B, Remme W, Zannad F, Neaton J, Martinez F, Roniker B, Bittman R, Hurley S, Kleiman J, Gatlin M, Eplerenone Post-Acute Myocardial Infarction Heart Failure Efficacy and Survival Study Investigators (2003). Eplerenone, a selective aldosterone blocker, in patients with left ventricular dysfunction after myocardial infarction. N Engl J Med.

[79] Tsutsui H, Momomura SI, Yamashina A, Shimokawa H, Kihara Y, Saito Y, Hagiwara N, Ito H, Yano M, Yamamoto K, Ako J, Inomata T, Sakata Y, Tanaka T (2019). Kawasaki Y; J-SHIFT Study Investigators. Efficacy and safety of ivabradine in Japanese patients with chronic heart failure-J-SHIFT study. Circ J.

[80] Abdul-Rahim AH, MacIsaac RL, Jhund PS, Petrie MC, Lees KR, McMurray JJ, On behalf the VICCTA-Heart Failure Collaborators (2016). Efficacy and safety of digoxin in patients with heart failure and reduced ejection fraction according to diabetes status: an analysis of the Digitalis Investigation Group (DIG) trial. Int J Cardiol.

[81] Fudim M, Sayeed S, Xu H, Matsouaka RA, Heidenreich PA, Velazquez EJ, Yancy CW, Fonarow GC, Hernandez AF, DeVore AD (2020). Representativeness of the PIONEER-HF clinical trial population in patients hospitalized with heart failure and reduced ejection fraction. Circ Heart Fail.

[82] Ito S, Satoh M, Tamaki Y, Gotou H, Charney A, Okino N, Akahori M, Zhang J (2015). Safety and efficacy of LCZ696, a first-in-class angiotensin receptor neprilysin inhibitor, in Japanese patients with hypertension and renal dysfunction. Hypertens Res.

[83] Vicent L, Ayesta A, Esteban-Fernández A, Gómez-Bueno M, de-Juan J, Díez-Villanueva P, Iniesta ÁM, Rojas-González A, Bover-Freire R, Iglesias D, García-Aguado M, Perea-Egido JA, Martínez-Sellés M (2019). Sex influence on the efficacy and safety of Sacubitril/valsartan. Cardiology..

[84] Gu J, Noe A, Chandra P, Al-Fayoumi S, Ligue-ros-Saylan M, Sarangapani R (2010). Pharmacokinetics and pharmacodynamics of LCZ696, a novel dual-acting angiotensin receptor-neprilysin inhibitor (ARNi). J Clin Pharmacol.

[85] Rahimtoola SH (2004). Digitalis therapy for patients in clinical heart failure. Circulation..

[86.•] Rathore SS, Wang Y, Krumholz HM. Sex-based differences in the effect of digoxin for the treatment of heart failure. N Engl J Med. 2002;347:1403–11. 10.1056/NEJMoa021266 12409542

[87] Lam CSP, Arnott C, Beale AL, et al. Sex differences in heart failure. Eur Heart J. 2019;40:3859–68. **The article focuses on differences in the outcome of digoxin use in heart failure with atrial fibrillation according to gender, with women having greater risk of death and worsening heart failure. Authors hypothesize an interaction between digoxin and hormone replacement therapy**. 10.1093/eurheartj/ehz835 31800034

